# Experimental Study of the Thermal Decomposition Properties of Binary Imidazole Ionic Liquid Mixtures

**DOI:** 10.3390/molecules27041357

**Published:** 2022-02-17

**Authors:** Fan Yang, Xin Zhang, Yong Pan, Hongpeng He, Yuqing Ni, Gan Wang, Juncheng Jiang

**Affiliations:** 1 College of Safety Science and Engineering, Nanjing Tech University, Nanjing 211816, China; 15895934959@163.com (F.Y.); xinzhang@njtech.edu.cn (X.Z.); hhp1002@outlook.com (H.H.); nyqforwork@outlook.com (Y.N.); wanggan@njtech.edu.cn (G.W.); jcjiang_njtech@163.com (J.J.); 2 School of Environment & Safety Engineering, Changzhou University, Changzhou 213164, China

**Keywords:** binary imidazole ionic liquid mixtures, thermal decomposition temperature, flash ignition temperature

## Abstract

Ionic liquids (ILs) have a wide range of applications, owing to their negligible vapor pressure, high electrical conductivity, and low melting point. However, the thermal hazards of ILs and their mixtures are also non-negligible. In this study, the thermal hazards of various binary imidazolium ionic liquids (BIIL) mixtures were investigated. The effects of parent salt components and molar ratios on the thermal decomposition temperature (*T_d_*) and flashpoint temperature (*T_f_*) are investigated. It is found that both *T_d_* and *T_f_* increase as the proportion of highly thermally stable components in BIIL mixtures increases. Furthermore, the decomposition process of BIIL mixtures can be divided into two stages. For most molar ratios, the *T_f_* of the BIIL mixtures is in the first stage of thermal decomposition. When the proportion of highly thermally stable components is relatively high, *T_f_* is in the second stage of thermal decomposition. The flammability is attributed to the produced combustible gases during the thermal decomposition process. This work would be reasonably expected to provide some guidance for the safety design and application of IL mixtures for engineering.

## 1. Introduction

Ionic liquids (ILs) are new green solvents composed of organic cations and organic or inorganic anions which possess excellent physical and chemical properties [1,2,3,4,5]. With the developments of applications, single ILs cannot meet specific application requirements [6]. However, ILs are well designed, which allows one to design functionalized IL mixtures by combining suitable anions and cations [7,8,9,10]. IL mixtures have been widely used in electrochemistry, carbon dioxide capture, biomass treatment, catalysis, extraction, and other fields [11,12,13,14,15,16,17,18,19].

In recent years, more and more researchers have focused on the thermal hazards of ionic liquid (IL) mixtures [20,21,22,23]. Many studies have pointed out that the thermal hazard of ILs is inextricably linked to the thermal decomposition properties [24,25,26,27,28,29]. Therefore, it is of great theoretical and practical significance to study the thermal decomposition properties of IL mixtures for safe design and safety control in industrial processes.

In previous studies, thermal analysis was used to clarify the thermal stability of IL mixtures. Therefore, it is necessary to analyze the thermal decomposition temperature (*T_d_*) of ionic liquid mixtures. Pinto et al. [30,31] and Altamash et al. [32] measured the *T_d_* of various ILs and their mixtures using a thermal gravimetric analyzer (TGA). They both found that the thermal stability of the mixtures increased as the ratio of the highly thermally stable components increased. Larriba et al. [33] investigated the effects of heating rates and mass losses on *T_d_* of [4bmpy][Tf_2_N] + [emim][EtSO_4_] and [4bmpy][Tf_2_N] + [emim][TFES]. They found that in most cases that the *T_d_* of the IL mixture was higher than that of the less thermally stable IL in the mixed system.

For a long time, fires and explosions of IL mixtures were easily overlooked due to the lack of the flash ignition temperature (*T_f_*) in the traditional sense. The *T_f_* is a key variable in characterizing the ignition risk of a liquid which needs to be tested. Liaw et al. [34] measured the *T_f_* of [C_6_mim][Cl] and [C_2_mim][NTf_2_] using a flash point meter. They found that the heat treatment temperature plays a role in the *T_f_* of IL. Liu et al. [35] took the ignition experiment of [BIM][NO_3_] using various experimental equipment. The FTIR spectrometry showed that the sample starts to exotherm at 170.0 °C. The amount of heat release is increased with the increased treatment temperature. The sample flashed between 177.2 and 190.4 °C. The combustible long-chain hydrocarbons during the pyrolysis process were identified by GC/MS. Li et al. [36] measured the *T_f_* of [BMIM][NO_3_] + [BMIM][BF_4_], [BIM][NO_3_] + [BMIM][BF_4_], [BIM][NO_3_] + [BMIM][NO_3_] and [BIM][BF_4_] + [BMIM][NO_3_] using flash point meter. They found that for a molar ratio of 5:5, the *T_f_* of the BIIL mixtures is close to the component with the lowest *T_f_* in the mixed system.

Through the literature review, we can initially understand that there is a link between the thermal decomposition and flash ignition behavior of ionic liquid mixtures. However, the studies in the literature have mainly focused on single ILs or a few binary IL mixtures in single molar ratios. There still lacks a systematic investigation of the thermal decomposition characteristics of IL mixtures. In this study, the thermal decomposition and flash combustion experiments of different BIIL mixtures were carried out. The effects of the parent salt composition and different molar ratios of IL mixtures on the *T_d_* and *T_f_* were investigated. Moreover, the link between these two temperatures was analyzed to reveal their thermal decomposition characteristics. These findings are expected to provide guidance for the safe design, synthesis, and application of IL mixtures.

## 2. Experimental

### 2.1. Materials for Thermal Decomposition Experiments

To analyze the influence of the components on the thermal decomposition temperature (*T_d_*), three cations with different substituents and four commonly used anions were selected. Thus, six kinds of imidazole ionic liquids (ILs) were selected. The ILs were purchased from Lanzhou Zhongke Kite Science and Industry Co., Ltd. (Lanzhou, China). They were stored in drying ovens to prevent the ILs from absorbing moisture from the air. The chemical structure and stability of the cations are shown in Table 1.

The ILs are mixed in molar ratios of 1:9, 3:7, 5:5, 7:3, and 9:1. The mixing rules were: one cation with different anions, one anion with different cations, and different anions with different cations. After excluding cases that the ILs are not mutually soluble or will react with each other, the results for 10 kinds of mixing ionic liquids were obtained, as shown in Table 2. To avoid absorbing water and other impurities present in the BIIL mixtures, the prepared BIIL mixtures were placed in a constant temperature drying oven at 60 °C for 24 h.

### 2.2. Materials for Flash Point Experiments

To analyze the influence of the components on the flash ignition temperature (*T_f_*), the same cations were chosen for this study as for the thermal decomposition experiments. For the anions, three less thermally stable anions and [NO_3_]^−^ were selected. Thus, six kinds of imidazole ionic liquids (ILs) were selected. The chemical structure and stability of cations are shown in Table 3. Like the mixing rule and methods as above, seven kinds of BIIL mixtures were obtained, as shown in Table 4.

### 2.3. Apparatus and Methods for Thermal Decomposition Experiments

The thermal gravimetric analyzer (TGA) is a thermal analysis process in which a sample’s quality is monitored in real-time according to time or temperature changes in a controlled environment. It is an effective way to measure the thermal stability of ILs. This experiment used the SDT-Q600 synchronous thermal analyzer (Waters Tech. Inc., Shanghai, China). The heating temperature was ranged from 30 °C to 500 °C, and the heating rate was set to 5 °C/min. The samples between 4 and 8 mg were placed in a 70 μL alumina crucible for TG experiments under a nitrogen atmosphere with a flow rate of 20 mL/min.

### 2.4. Apparatus and Methods for Flash Ignition Experiments

The *T_f_* of BIIL mixtures was measured using a FP CC-420A trace continuous closed-end flash point meter (Yang Yi Tech. Inc., Hangzhou, China). Due to the high viscosity of most ILs, these experiments used the ASTM D93A method. The method is widely used for viscous liquids and enables accurate determination of the flash ignition temperature of ILs [32]. Based on this method, the heating temperature was ranged from 40 °C to 370 °C, the heating rate was set to 5.5 °C/min ± 0.5 °C/min, the flashpoint meter ignition frequency was 1 °C, and the ignition time was 19 ms ± 2 ms, the stirring rate was 110 r/min. For ILs, the ASTM D93A standard allows for an experimental error of ±2.5 °C. To reduce errors, each test was averaged by three valid *T_f_* data.

## 3. Results and Discussion

### 3.1. The Thermal Decomposition Temperature of BIIL Mixtures

**Figure 1:**
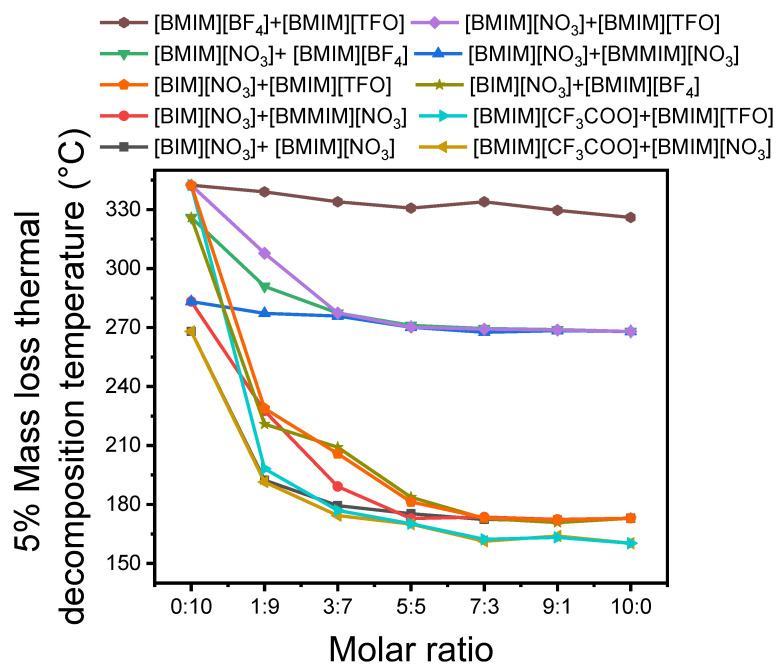
The *T_d5%_* as a function of the molar ratio of BIIL mixtures with different components.

In this study, the 5% onset decomposition temperature (*T_d5%_*) is used as a parameter to characterize the thermal stability of binary imidazole ionic liquids (BIIL) mixtures. The *T_d5%_* thermographs for a series of BIIL mixtures are shown in Figure 1. As the molar ratio increases, the *T_d5%_* decreases rapidly, then decreases slowly, and finally tends to be constant. It is found that when the proportion of the highly thermally stable components is lower, the thermal stability of BIIL mixtures is consistent with the less thermally stable components. As the proportion of the highly thermally stable components rises ≥0.5, the thermal stability of BIIL mixtures gradually increases. These findings are consistent with those obtained from the experiments of Pinto et al [30].

**Figure 2:**
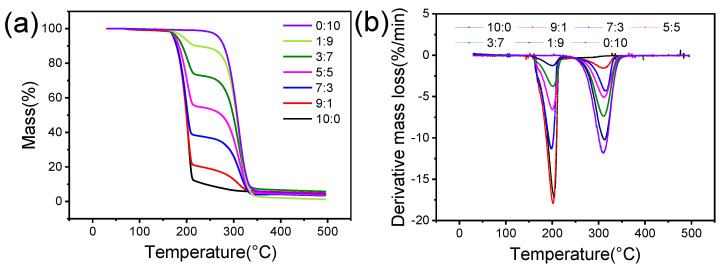
(**a**) TG and (**b**) DTG curves of BIIL mixtures of [BIM][NO_3_] + [BMIM][NO_3_].

[BIM][NO_3_] + [BMIM][NO_3_] is used as an example to analyze the *T_d_* at different molar ratios, and its thermal decomposition curve is shown in Figure 2a. It is found that the thermal decomposition process of the [BIM][NO_3_] + [BMIM][NO_3_] shows two stages. The thermal behaviors of these mixtures are similar to that of the parent salt. The [BIM][NO_3_] + [BMIM][NO_3_] decomposes hardly at all until 172.1 °C. Then, it decomposes suddenly when the temperature rises to between 172.1 and 283.2 °C. The *T_d_* gradually increases with the increasing molar ratio.

The derivative thermogravimetric (DTG) curves were also plotted to investigate the thermal decomposition process further, as shown in Figure 2b. The DTG curve shows the two-stage thermal decomposition process more clearly. As the molar ratio increases, the rate of the first-stage thermal decomposition of the [BIM][NO_3_] + [BMIM][NO_3_] increases, and the peak temperature corresponding to the thermal decomposition decreases. 

Compared with the *T_d5%_* with different molar ratios, it is found that the *T_d5%_* of the BIIL mixtures remain essentially constant at higher molar ratios, which is consistent with the *T_d5%_* of the less thermally stable components. For the ratios of 9:1 and 7:3, the *T_d5%_* of the mixture was 172.1 °C and 172.3 °C. They are closer to the *T_d5%_* for pure [BIM][NO_3_] (173.0 °C). However, as the proportion of [BMIM][NO_3_] increases to the ratio ≥0.5, the *T_d5%_* for the whole system gradually increases. For the ratios of 5:5, 3:7, and 1:9, the *T_d5%_* are 175.3 °C, 179.4 °C, and 192.4 °C, respectively, and the thermal stability is gradually enhanced.

### 3.2. The Flash Ignition Temperatures of BIIL Mixtures

**Figure 3:**
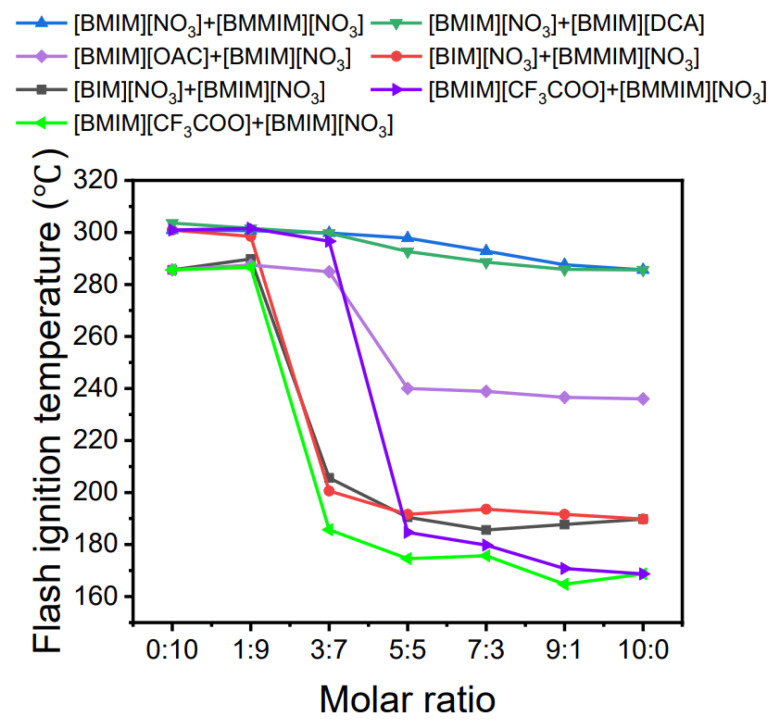
The *T_f_* as a function of the molar ratio of BIIL mixtures.

The *T_f_* measured for the BIIL mixtures are shown in Figure 3. The *T_f_* gradually decreases as the molar ratio increases. When the proportion of less thermally stable components is high, the *T_f_* of the BIIL mixtures changes slowly. When the molar ratio of the mixtures is less than 3:7, the *T_f_* of the BIIL mixtures show a significant upward trend.

**Figure 4:**
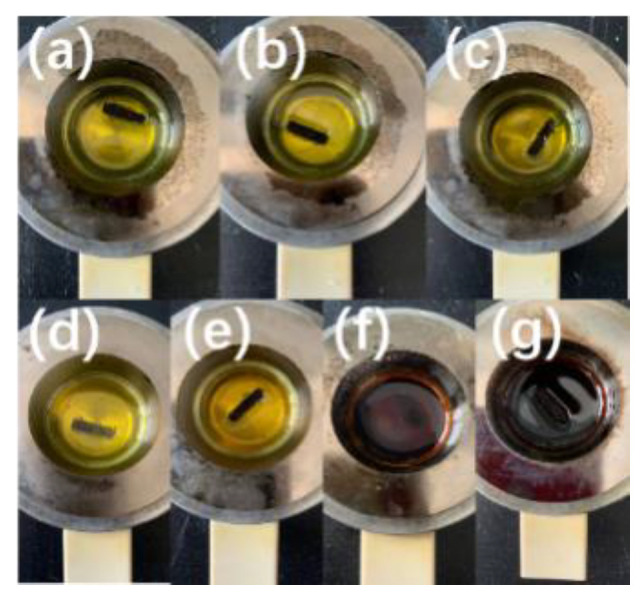
Color change before and after the *T_f_* experiments of the [BIM][NO_3_] + [BMIM][NO_3_] mixtures with molar ratio of (**a**) 10:0, (**b**) 9:1, (**c**) 7:3, (**d**) 5:5, (**e**) 3:7, (**f**) 1:9, and (**g**) 0:10.

**Figure 5:**
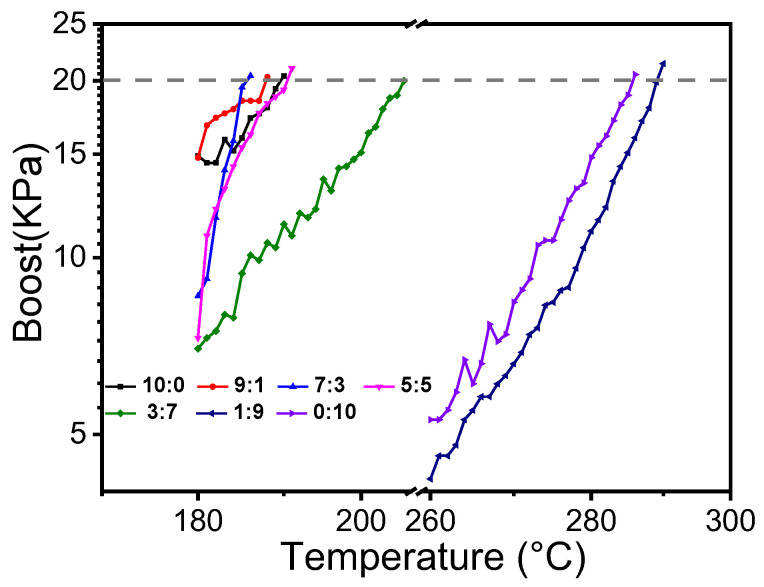
*T_f_*-boost curves of the [BIM][NO_3_] + [BMIM][NO_3_] mixtures with various molar ratio.

In the flash ignition experiments, the colour and viscosity of the BIIL mixtures changed obviously. This indicates that the chemical properties of the BIIL mixtures were changed during the heating process. These changes were most likely caused by the partial thermal decomposition of the BIIL mixtures. This is consistent with the conclusion reported by Liu et al [35]. In the case of [BIM][NO_3_] + [BMIM][NO_3_], as shown in Figure 4a–g, the molar ratios of 10:0, 9:1, 7:3, 5:5, 3:7, 1:9, and 0:10 were experimentally changed from a colourless, transparent liquid to a yellow liquid. It can be inferred that the flash ignition of [BIM][NO_3_] + [BMIM][NO_3_] in the ratios of 9:1, 7:3, 5:5, and 3:7 is caused by the component [BIM][NO_3_]. In contrast, the BIIL mixtures with the molar ratio of 0:10 and 1:9 show a black and a denser texture, which may be due to the carbonization of the ionic liquid at high temperatures. These observations suggest that the combustion of ionic liquid mixtures may depend on their thermal decomposition process. It is different from the traditional definition of a flammable liquid undergoing natural vaporisation. Figure 5 further validates this conclusion.

In general, traditionally flammable liquids can evaporate under heated conditions and the system pressure will suddenly and dramatically increase. However, unlike traditionally flammable liquids, IL mixtures have very low vapor pressures and virtually no evaporation under heating. Figure 5 shows that the system pressure of [BIM][NO_3_] + [BMIM][NO_3_] gradually increases with the temperature rise in the flash ignition experiment. Thus, we can infer that the pressure change in the plot is caused by the thermal decomposition of the BIIL mixture. As the temperature rises, the combustible gases produced by thermal decomposition are flash. Additionally, from Figure 5, it is found that *T_f_* is related to the parent salt and its molar ratio. With the molar ratio of 9:1, 7:3, and 5:5, the *T_f_* of [BIM][NO_3_] + [BMIM][NO_3_] is 187.7 °C, 185.6 °C, and 190.5 °C, respectively, which are closer to the *T_f_* of [BIM][NO_3_] (189.8 °C). As the proportion of the highly thermally stable component of [BMIM][NO_3_] increases to the molar ratio of 3:7, the *T_f_* of the mixtures increases slightly to 205.6 °C. When the [BMIM][NO_3_] has a high proportion of 1:9, the *T_f_* of the mixtures is 289.8 °C, which is closer to the *T_f_* of [BMIM][NO_3_] (285.6 °C). These results indicate that the *T_f_* of BIIL mixtures depends on the thermal decomposition process. It occurs in two separate thermal decomposition stages.

### 3.3. Correlation between Thermal Decomposition and the Flash Ignition Temperatures of BIIL Mixtures

**Figure 6:**
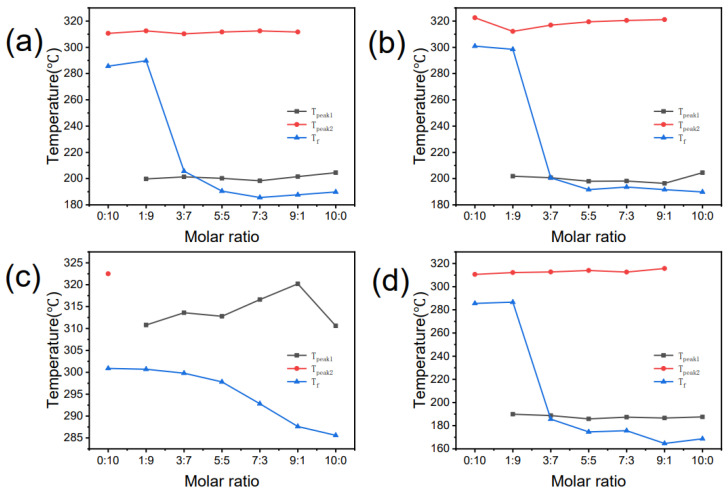
The first and second peak temperature of thermal decomposition (*T_peak1_*, *T_peak2_*) as well as *T_f_* for (**a**) [BIM][NO_3_] + [BMIM][NO_3_], (**b**) [BIM][NO_3_] + [BMMIM][NO_3_], (**c**) [BMIM][NO_3_] + [BMMIM][NO_3_], (**d**) [BMIM][CF_3_COO] + [BMIM][NO_3_].

To further analyze the relationship between flash ignition and the two stages of thermal decomposition, we have plotted Figure 6a–d. It shows the *T_f_* of BIIL mixtures, the peak temperatures corresponding to the first and second thermal decomposition stages (*T_peak1_*, *T_peak2_*), respectively. As shown in Figure 6a,b,d, the *T_f_* of BIIL mixtures shows the same tendency. It decreases sharply and then slowly as the molar ratio increases. In most cases, the *T_f_* is between the *T_peak1_* and *T_peak2,_* when the molar ratio is lower than 3:7. However, when molar ratios are higher than 3:7, the *T_f_* of the BIIL mixtures is lower than the *T_peak1_*. As shown in Figure 6c, for ratios higher than 0:10, [BMIM][NO_3_] + [BMMIM][NO_3_] undergoes only the first stage of thermal decomposition, and its *T_f_* is all below *T_peak_*.

Taking [BIM][NO_3_] + [BMIM][NO_3_] as an example (Figure 6a), the variation of *T_f_* and *T_d_* are analyzed. For a molar ratio of 10:0, the *T_f_* is 189.8 °C, which is slightly higher than its *T_d5%_* of 173.0 °C and slightly lower than the *T_peak1_* of 204.5 °C. For the molar ratio of 0:10, the *T_f_* is 285.6 °C, which is slightly higher than its *T_d5%_* of 268.0 °C and slightly lower than the *T_peak2_* of 310.6 °C. For the molar ratios of 9:1, 7:3, and 5:5, the *T_f_* are 187.7 °C, 185.6 °C, and 190.5 °C, respectively, which are slightly higher than their *T_d5%_* of 172.1 °C, 172.3 °C, and 175.3 °C, and slightly lower than their *T_peak1_* of 201.5 °C, 198.3 °C, and 200.2 °C, respectively. This phenomenon indicates that the BIIL mixtures need to undergo thermal decomposition to produce a sufficient concentration of combustible gases before flash ignition occurs. For a molar ratio of 3:7, the *T_f_* is 205.6 °C, slightly higher than its *T_d5%_* of 179.4 °C and close to the *T_peak1_* of 201.3 °C. This phenomenon indicates that when the component with lower thermal stability is under-represented, only a continuous increase in temperature to the maximum thermal decomposition rate can produce a sufficient concentration of combustible gases and thus flash ignition. However, when the molar ratio is 1:9, *T_f_* is 289.8 °C, which is significantly higher than its *T_d5%_* of 192.4 °C and slightly lower than its *T_peak2_* of 312.5 °C. It is due to the lower proportion of less thermally stable components in the mixture at this ratio. Thus, the mixtures are thermally stable and only flash ignite in the second thermal decomposition stage. So *T_f_* is also significantly higher than *T_d5%_* (268.0 °C) for pure [BMIM][NO_3_]. In Figure 6b–d, we can observe the same phenomenon for the other BIIL mixtures. Of these, only a single *T_peak_* is observed for [BMIM][NO_3_] + [BMMIM][NO_3_] (Figure 6c). This phenomenon is due to the thermal decomposition temperatures of [BMIM][NO_3_] and [BMMIM][NO_3_] are close to each other, so the thermal decomposition process of the mixture shows a one stage thermal decomposition, but its *T_f_* still lies between the *T_d5%_* and *T_peak_*.

From the above experimental phenomena, we can conclude that, at most ratios, the less thermally stable components of BIIL mixtures are the first to decompose at high temperatures thermally. Hence, its flash ignition depends on the first thermal decomposition stage. As the thermal decomposition rate gradually increases, the thermal decomposition of BIIL mixtures produces sufficient concentrations of combustible gases, and flash ignition occurs when they come into contact with flame. Therefore, the *T_f_* of BIIL mixtures lies between the *T_d5%_* and the *T_peak1_*. As the proportion of thermally stable components increases, the *T_f_* of the BIIL mixtures increases and gradually approaches the *T_peak1_*. However, when the proportion of thermally stable components is ≥0.7, the first stage of the thermal decomposition of the BIIL mixtures does not produce sufficient concentrations of combustible gases to cause flash ignition, which occurs in the second stage of thermal decomposition. The *T_f_* is significantly higher than the *T_d5%_* of the mixtures and lies between the *T_d5%_* of the highly thermally stable component and the *T_peak2_* of the second thermal decomposition stage of the mixtures.

## 4. Conclusions

The effects of component and molar ratio of binary imidazole ionic liquid (BIIL) mixtures on thermal decomposition temperature (*T_d_*) and the flash ignition temperature (*T_f_*) are systematically investigated. The main conclusions can be summarized as below:

(1) When the proportion of less thermally stable components is high, the *T_d_* of the BIIL mixture is consistent with that of the less thermally stable components, which might promote intense combustible and toxic gases production. When the proportion of highly thermally stable components increases above 0.5, the *T_d_* of the mixtures gradually increases. That means, the thermal stability of the mixtures increases;
(2) When the proportion of less thermally stable components is high, the *T_f_* of the mixture is close to that of the less thermally stable components. This situation can easily induce a fire and explosion accident. As the proportion of the highly thermally stable components increases, the Tf of the mixture also increases. It is accordingly lower in thermal risk. When the proportion of the highly thermally stable component is ≥0.7, the *T_f_* of the mixture is consistent with the *T_f_* of the highly thermally stable component;
(3) In the cases of most molar ratios, the flash ignition of BIIL mixtures depends on the first stage of the thermal decomposition process. As the proportion of highly thermally stable components increases, the *T_f_* of the IL mixtures increases. When the highly thermally stable component has a high proportion ≥ 0.7, the *T_f_* is in the second stage of the thermal decomposition process.

In summary, to improve the safety of ionic liquid mixtures, the parent salt type and the molar ratio of the mixture should be fully considered when designing the ionic liquid mixtures.

## Figures and Tables

**Table 1:** Structures and formulas of ILs for *T_d_* experiments.

No.	Name	Abbreviation	Cation		Anion Structure
			Structure	Stability	
1	1-butyl-3-methylimidazolium tetrafluoroborate	[BMIM][BF_4_]	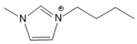	Stable	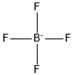
2	1-butyl-3-methylimidazolium trifluoromethanesulfonate	[BMIM][TFO]	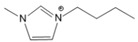	Stable	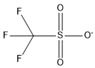
3	1-butyl-3-methylimidazolium nitrate	[BMIM][NO_3_]	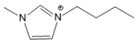	Stable	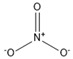
4	1-butyl-3-methylimidazolium trifluoroacetate	[BMIM][CF_3_COO]	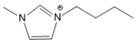	Stable	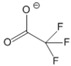
5	1-butyl-imidazolium nitrate	[BIM][NO_3_]	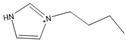	Unstable	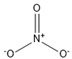
6	1-butyl-2,3-dimethylimidazolium nitrate	[BMMIM][NO_3_]	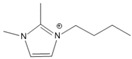	Very stable	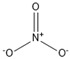

**Table 2:** Composition of the BIIL mixtures for *T_d_* experiments.

No.	BIIL Mixtures	Cation	Anion
1	[BIM]_x_[BMIM]_(1−x)_[NO_3_]_x_	[BIM]^+^, [BMIM]^+^	[NO_3_]^−^
2	[BIM]_x_[BMMIM]_(1−x)_[NO_3_]_x_	[BIM]^+^, [BMMIM]^+^	[NO_3_]^−^
3	[BMIM]_x_[BMMIM]_(1−x)_[NO_3_]_x_	[BMIM]^+^, [BMMIM]^+^	[NO_3_]^−^
4	[BMIM][NO_3_]_x_[TFO]_(1−x)_	[BMIM]^+^	[NO_3_]^−^, [TFO]^−^
5	[BMIM][NO_3_]_x_[BF_4_]_x_	[BMIM]^+^	[NO_3_]^−^, [BF_4_]^−^
6	[BMIM][NO_3_]_x_[CF_3_COO]_x_	[BMIM]^+^	[NO_3_]^−^, [CF_3_COO]^−^
7	[BMIM][BF_4_]_(1−x)_[TFO]_(1−x)_	[BMIM]^+^	[BF_4_]^−^, [TFO]^−^
8	[BMIM][CF_3_COO]_x_[TFO]_(1−x)_	[BMIM]^+^	[CF_3_COO]^−^, [TFO]^−^
9	[BIM]_x_[BMIM]_(1−x)_[NO_3_]_y_[TFO]_(1−y)_	[BIM]^+^, [BMIM]^+^	[NO_3_]^−^, [TFO]^−^
10	[BIM]_x_[BMIM]_(1−x)_[NO_3_]_y_[BF_4_]_(1−y)_	[BIM]^+^, [BMIM]^+^	[NO_3_]^−^, [BF_4_]^−^

**Table 3:** Structures and formulas of ILs for *T_f_* experiments.

No	Name	Abbreviation	Cation		Anion Structure
			Structure	Stability	
1	1-butyl-imidazolium nitrate	[BIM][NO_3_]	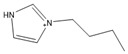	Unstable	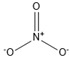
2	1-butyl-3-methylimidazolium nitrate	[BMIM][NO_3_]	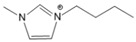	Stable	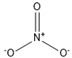
3	1-butyl-2,3-dimethylimidazolium nitrate	[BMMIM][NO_3_]	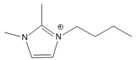	Very stable	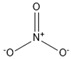
4	1-butyl-3-methylimidazolium acetate	[BMIM][OAC]	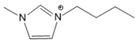	Stable	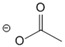
5	1-butyl-3-methylimidazolium dicyanamide	[BMIM][DCA]	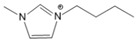	Stable	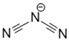
6	1-butyl-3-methylimidazolium trifluoroacetate	[BMIM][CF_3_COO]	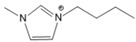	Stable	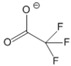

**Table 4:** Composition of the BIIL mixtures for *T_f_* experiments.

No	BIIL Mixtures	Cation	Anion
1	[BIM]_x_[BMIM]_(1−x)_[NO_3_]_(1−x)_	[BIM]^+^, [BMIM]^+^	[NO_3_]^−^
2	[BIM]_x_[BMMIM]_(1−x)_[NO_3_]_(1−x)_	[BIM]^+^, [BMMIM]^+^	[NO_3_]^−^
3	[BMIM]_x_[BMMIM]_(1−x)_[NO_3_]_(1−x)_	[BMIM]^+^, [BMMIM]^+^	[NO_3_]^−^
4	[BMIM][NO_3_]_x_[OAC]_(1−x)_	[BMIM]^+^	[NO_3_]^−^, [OAC]^−^
5	[BMIM][NO_3_]_x_[DCA]_(1−x)_	[BMIM]^+^	[NO_3_]^−^, [DCA]^−^
6	[BMIM][NO_3_]_x_[CF_3_COO]_(1−x)_	[BMIM]^+^	[NO_3_]^−^, [CF_3_COO]^−^
7	[BMIM]_x_[BMMIM]_(1x)_[NO_3_]_y_[CF_3_COO]_(1−y)_	[BMIM]^+^, [BMMIM]^+^	[NO_3_]^−^, [CF_3_COO]^−^

## Data Availability

The data presented in this study are available on request from the corresponding author.

## Sample Availability

Samples of the ionic liquids and their mixtures in the article are available from the authors.
